# Epigenetic control of pancreatic cancer metastasis

**DOI:** 10.1007/s10555-023-10132-z

**Published:** 2023-09-02

**Authors:** Lukas Krauß, Carolin Schneider, Elisabeth Hessmann, Dieter Saur, Günter Schneider

**Affiliations:** 1 Department of General, Visceral and Pediatric Surgery, University Medical Center Göttingen, 37075 Göttingen, Germany; 2 Department of Gastroenterology, Gastrointestinal Oncology and Endocrinology, University Medical Center Göttingen, 37075 Göttingen, Germany; 3 Clinical Research Unit 5002, KFO5002, University Medical Center Göttingen, 37075 Göttingen, Germany; 4 CCC-N (Comprehensive Cancer Center Lower Saxony), 37075 Göttingen, Germany; 5 Institute for Translational Cancer Research and Experimental Cancer Therapy, Technical University Munich, 81675 Munich, Germany; 6 German Cancer Research Center (DKFZ) and German Cancer Consortium (DKTK), 69120 Heidelberg, Germany

**Keywords:** Pancreatic cancer metastasis, KDM, KMT, HAT, HDAC, Epigenetic

## Abstract

Surgical resection, when combined with chemotherapy, has been shown to significantly improve the survival rate of patients with pancreatic ductal adenocarcinoma (PDAC). However, this treatment option is only feasible for a fraction of patients, as more than 50% of cases are diagnosed with metastasis. The multifaceted process of metastasis is still not fully understood, but recent data suggest that transcriptional and epigenetic plasticity play significant roles. Interfering with epigenetic reprogramming can potentially control the adaptive processes responsible for metastatic progression and therapy resistance, thereby enhancing treatment responses and preventing recurrence. This review will focus on the relevance of histone-modifying enzymes in pancreatic cancer, specifically on their impact on the metastatic cascade. Additionally, it will also provide a brief update on the current clinical developments in epigenetic therapies.

## Background

Pancreatic ductal adenocarcinoma (PDAC) is the third leading cause of cancer-related death in the USA and has the lowest 5-year survival rate of all cancer types, with only 12% [1]. The current standard of care for most patients involves modestly effective combination chemotherapies, such as a modified FOLFIRINOX regimen or gemcitabine and nanoparticle albumin-bound paclitaxel [2]. By the time of diagnosis, around 50% of patients have already developed metastasis, while around 30 to 35% have locally advanced, unresectable tumors. Only 10–20% of patients harbor localized tumors, which allows curative-intent resections in the context of neo-adjuvant/adjuvant therapies [1–3]. However, it is important to note that even resectable PDACs are likely to be disseminated and should be treated as a systemic disease. Genetically engineered mouse models have shown inflammation-driven epithelial to mesenchymal transition (EMT) and dissemination early in the disease [4]. Furthermore, the metastatic probability of a 2-cm-sized PDAC was calculated to be 73%, and 94% for 3-cm-sized cancer, respectively. Considering the average diameter of PDAC at surgery is 3–4 cm underscores the need for neo-adjuvant/adjuvant therapies even for resectable patients [5]. Furthermore, these data highlight the demand for a detailed understanding of the metastatic cascade.

Tumor initiation in PDAC is predominantly caused by *KRAS* mutations, occurring in approximately 90% of patients. In addition to *KRAS*, major genetic alterations occur in *TP53*, *CDKN2A*, and *SMAD4* in up to 80%, 26%, and 25% of patients, respectively [6–8]. The sequencing of primary tumors and matched metastatic tissues has demonstrated high concordance of driver gene mutations [9, 10]. However, increased gene dose of the *KRAS* oncogene [11, 12] as well as the amplification or activation of *MYC* [13, 14] have been linked to tumor progression and metastasis. When considering frequencies, e.g., major KRAS imbalances in favor of the mutant allele occur in 4% of primary tumors compared to 29% in metastatic disease [12], it is probable that multiple pathways contribute. Furthermore, the described concordance of mutations in metastasizing and non-metastasizing PDACs underscores the contribution of non-genetic, transcriptional reprogramming and epigenetic plasticity to the metastatic cascade of PDAC. Early in carcinogenesis, differential chromatin modules identify heterogeneous cell-states primed for later emerging tumor cell fates [15]. This emphasizes the significance of epigenetic plasticity in the biology of PDAC. Consistently, large chromatin domains are reprogrammed during tumor progression [10], and a growing set of transcription factors (TF), such as Forkhead family TFs [16, 17], BLIMP1 [18], RUNX3 [19], ZEB1 [20], PRRX1 [21], FOSL1 [22, 23], or deltaNp63 [24, 25], have been associated with dedifferentiation and metastasis.

The liver and lungs are the most commonly affected distant organ sites where metastasis of PDAC occurs. The molecular patterns responsible for this organ site specificity are currently under investigation [7, 26–28]. Recent efforts to subtype PDAC have identified two classical (A/B) and two basal-like (A/B) subtypes, as well as a hybrid subtype [12]. The classical subtypes are characterized by a slightly better prognosis. These subtypes exhibit an enriched expression of the pancreatic differentiation factor *GATA6* and an increased occurrence of *SMAD4* alterations. In contrast, basal-like subtypes are characterized by an enrichment of EMT and TGF-beta signatures as well as an increased occurrence of *TP53* mutations and complete loss of *CDKN2A* [12]. The most aggressive and chemoresistant subtype, basal-like A, displays the highest frequency of intact SMAD4 [12]. As basal-like A PDACs exhibit a higher probability of metastasis and are enriched in EMT and TGF-beta signatures, it raises the question of how the tumor-suppressive arm of TGF-beta signaling is overcome in SMAD4 wild-type PDACs, a question addressed by several recent studies [29–31]. While EMT and TGF-beta signaling are tightly linked and promote a more aggressive phenotype, an increased understanding of cellular plasticity has promoted the concept of partial EMT (p-EMT) as a more plastic, reversible morphological state [32]. The characteristics of p-EMT fundamentally differ from the classical EMT processes. Particularly, p-EMT might be mediated independently of TGF-beta signaling. Observations of circulating tumor cells (CTC) also discovered that cells in a p-EMT state often disseminate in clusters whereas fully mesenchymal cells mostly travel as individual cells in the vascular system. On closer inspection, pancreatic cancer patients with a higher number of CTCs in the p-EMT state displayed a shorter progression-free survival [33].

**Fig. 1:**
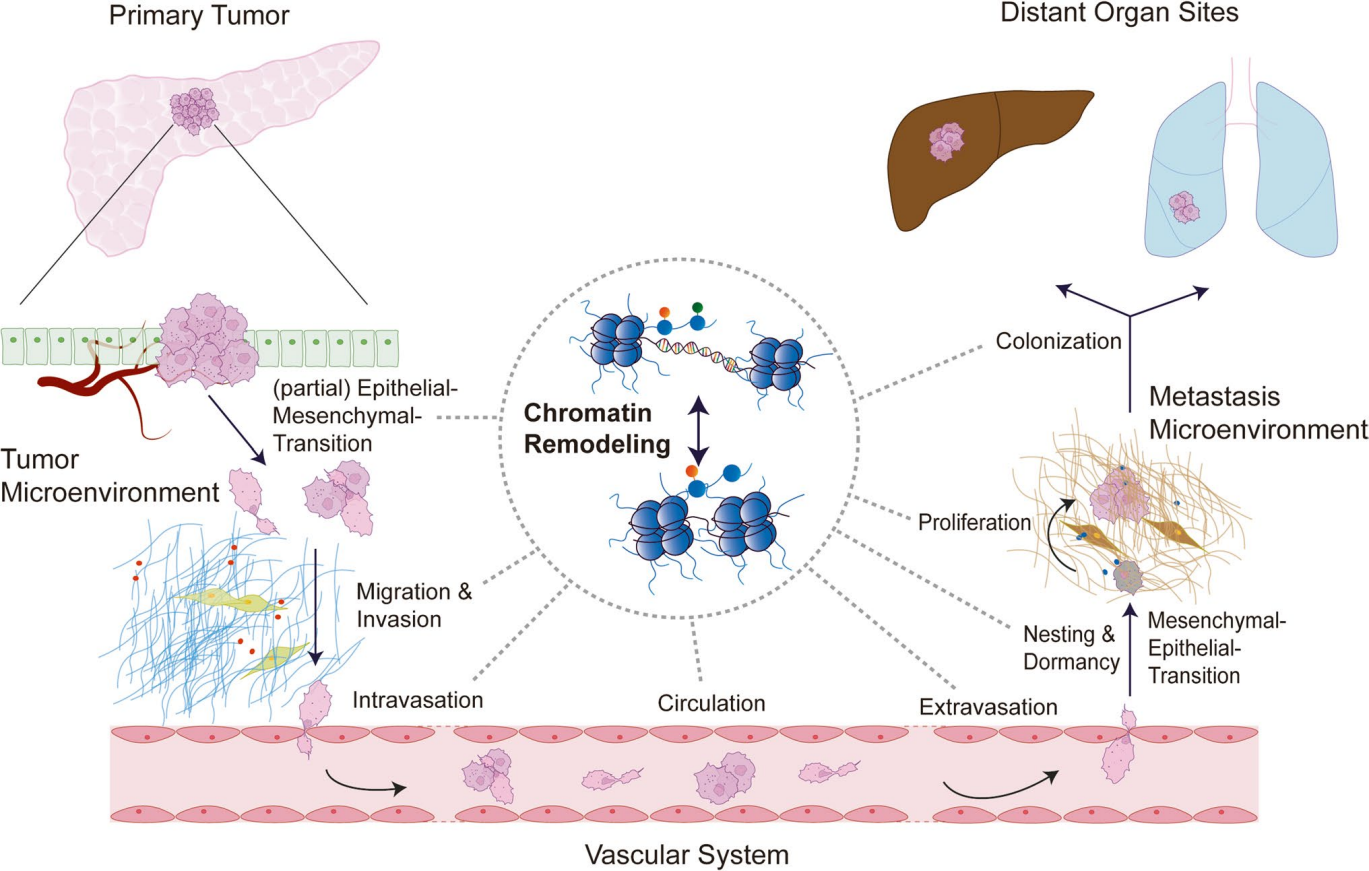
PDAC cells undergo chromatin remodeling during metastatic processes. Disseminating tumor cells constantly change their chromatin landscape to adapt to the challenges during the metastatic process. These include EMT, migration and invasion, intravasation, anchorage-independent survival in the circulation, extravasation, nesting and dormancy, MET, proliferation, and distant organ colonization

The cellular, transcriptional, and epigenetic plasticity of cancer cells [34, 35] is especially relevant in PDAC [36]. Cellular plasticity processes induced by intracellular or extracellular cues can occur within just a few days *in vivo*, completely changing the metastatic capabilities. In murine autochthonous PDAC, genetic manipulation of the bone morphogenetic protein (BMP) signaling inhibitor Gremlin 1 (GREM1) *in vivo* converted epithelial differentiated PDACs to undifferentiated mesenchymal PDAC, which was linked to a distinct increase in metastatic frequency [37]. Mesenchymal cells produce GREM1, which acts in a paracrine fashion to block BMP signaling and the induction of the EMT transcription factors Snail family transcriptional repressor 1/2 (SNAI1/2) in epithelial cells. These findings illustrate how PDACs use signaling-linked transcriptional reprogramming to shape intra-tumoral heterogeneity and metastasis [37]. Additionally, these findings suggest that tumor cells require highly adaptable and fluidic capabilities to pass through the complete metastatic cascade (Fig. 1), which is known to be tightly controlled by transcriptional networks and the epigenetic machinery [10, 38–41]. The support for this note is based on an additional study that aimed to identify metastasis drivers in a large cohort of molecularly characterized cancer patients. The study involved 1779 PDAC patients and compared primary tumors with metastatic tumors. Alongside *CDKN2A* and *SMAD4* deletions, the TGF-beta signaling pathway, cell-cycle signatures, and the epigenetic pathway were linked to metastasis in PDAC [7]. These epigenetic pathways encompass a variety of mechanisms that contribute to cellular plasticity and metastasis. For instance, elegant studies identified hypomethylated DNA signatures in PDAC cells that coordinate the reorganization of the microenvironment via Interferon-alpha (IFNα) signaling and link DNA methylation to the more aggressive basal-like subtype [42]. Beyond DNA methylation, the genome’s three-dimensional architecture, non-coding RNAs, and histone modifications present other relevant ways of epigenetic contribution to plasticity [43]. Here we focus on recent insights into the contribution of histone-modifying enzymes to metastasis and their potential as a therapeutic target. We will spotlight lysine modifiers.

## Histone modifiers in PDAC metastasis

The dissemination of tumor cells is a complex process that involves several stages in the formation of metastases and requires continuous adaptation [35] (Fig. 1). Starting with challenges in nutrient supply during tumor growth, disseminating cells are forced to survive the lack of adhesion during their travel to distant organs while constantly evading immune surveillance. Once tumor cells arrive at a distant organ site, they must re-establish cell growth in a completely new (micro-) environment [3, 41]. Unlike fixed genomic alterations, the reversibility of histone marks grants cancer cells the capability to undergo dynamic shifts in the epigenetic landscape, which is relevant to cope with challenges posed by the metastatic cascade [41]. Histone-modifying enzymes, such as histone deacetylases (HDACs), histone acetyltransferases (HATs), as well as lysine methyltransferases (KMTs) and lysine demethylases (KDMs), play crucial roles in conferring adaptability to disseminating cells. Notably, the reprogramming of enhancers by epigenetic modifications at histones during metastatic transition and as means of drug resistance is a commonly observed phenomenon [16, 44]. This is furthermore illustrated by a previous study, which demonstrated the linkage between metabolic changes in primary PDAC tumors and metastatic sites, mediated by a global reorganization of the epigenetic landscape [10]. Additionally, the study showed the collective changes in histone methylation and acetylation as well as their interdependence, demonstrating the relevance of the epigenetic machinery for PDAC metastasis.

**Fig. 2:**
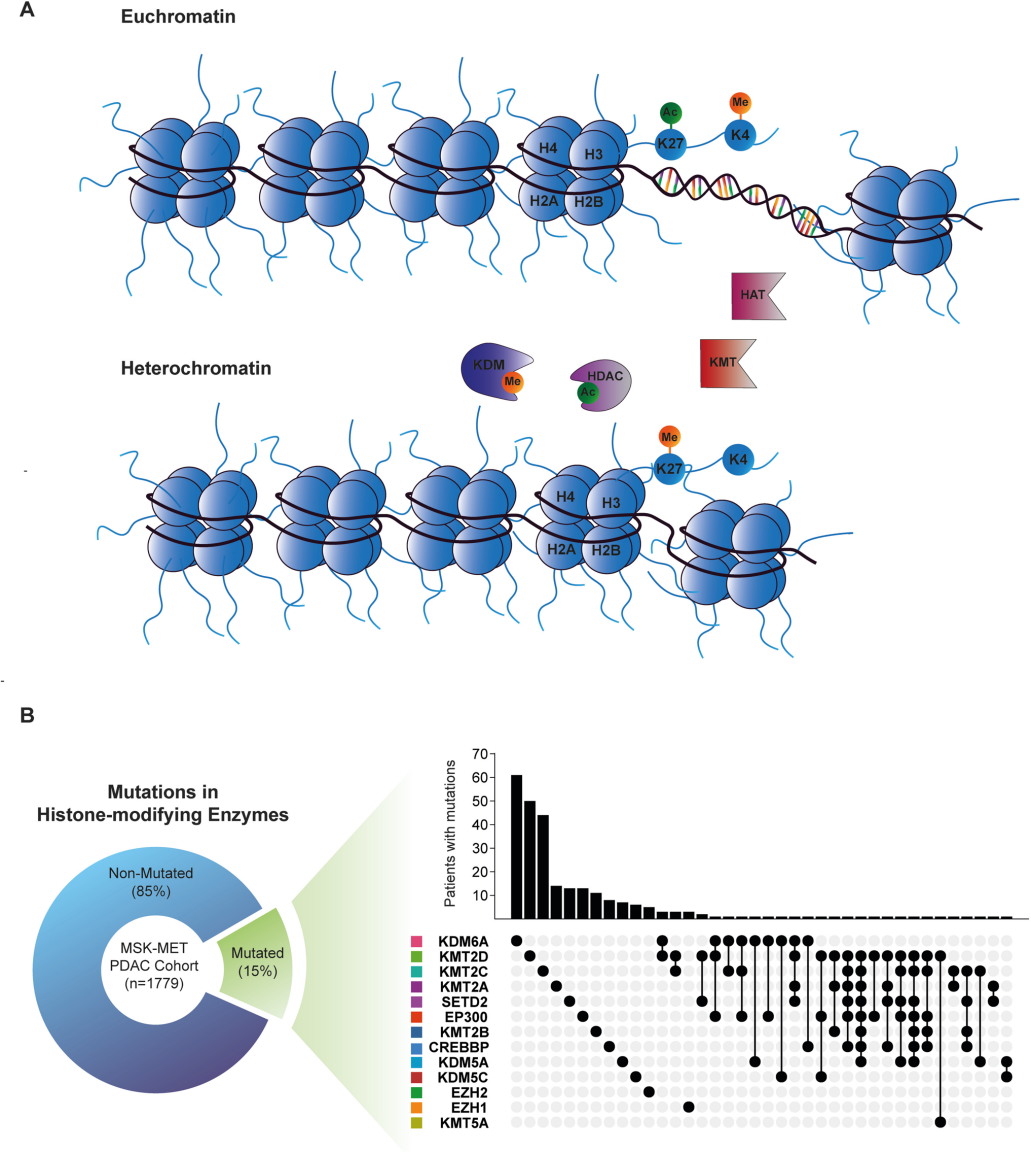
Epigenetic readers and writers in PDAC. **A** HATs and KMTs induce euchromatin by acetylation of H3K27 and methylation of H3K4. HDACs and KMT deacetylate and methylate H3K27 to induce heterochromatin. KDMs remove methylations at H3K4 or H3K27, respectively. **B** (Left): 15% (*n*=266) of PDAC patients in the MSK MetTropsim PDAC cohort (*n*=1779) display mutations in histone-modifying enzymes (Right): Upset-plot displaying the number of patients with single and multiple (connected dots) mutations in histone-modifying enzymes. Data and plots were in part extracted from cBioPortal [73, 74]

Histone modifications, such as acetylation or methylation, can coordinate diverse transcriptional dynamics depending on their genomic location and the modified histone residue. For example, H3K4 trimethylation (H3K4me3) along with H3K27 acetylation (H3K27ac) are typically observed at open and active promoter sites [45–50]. On the other hand, H3K27 trimethylation (H3K27me3) signifies heterochromatin and causes transcriptional repression (Fig. 2a) [51]. H3K27ac at promotor and enhancer sites can lead to the binding of bromodomain and extraterminal domain (BET) proteins that act as scaffolds for the recruitment of the transcription elongation machinery as well as transcription factors [52–54]. The family of BET proteins, often referred to as “epigenetic readers,” includes bromodomain-containing proteins (BRD) 2-4 as well as the testis-specific BRDT. Particularly, BRD4 has been well characterized as it recruits positive transcriptional elongation factor (P-TEFb) to acetylated transcriptional start sites and promotes transcriptional elongation [55]. Numerous studies showed a causal relationship with expression of oncogene c-MYC and BRD4, including pancreatic cancer [56]. For instance, inhibition of BRD4 by shRNA or BET-inhibitor (BETi) JQ1 reduced MYC protein levels in PDAC cell lines [56]. Additionally, JQ1 effectively inhibited tumor growth in primary pancreatic cancer xenografts in MYC high groups, providing a stratification strategy for MYC-dependent tumors [57]. Furthermore, BRD4 is frequently recruited by enhancer-binding factors such as yes-associated protein (YAP)/PDZ-binding motif (TAZ) to enable enhancer-promoter interaction in malignancies [58]. Recent studies identified the transcriptional control of receptor tyrosine kinase-like orphan receptor 1 (ROR1) via YAP-BRD4 enhancer interactions and associated high ROR1 expression with a partial-EMT state. In this study, ROR1-induced tumorigenesis and its knockdown reduced metastasis formation in orthotopically transplanted mice [59]. BET protein-dependent enhancer-promoter interaction also influences subtype identities in PDAC. Elegant studies discovered that BET family member bromodomain containing 4 (BRD4) maintained cJUN expression which resulted in the recruitment of TNF-alpha positive macrophages via the BRD4-cJUN/AP1-CCL2 axis and subsequent TNF-alpha-mediated classical-to-basal subtype switch [60]. In addition, cJUN collaborates with progenitor transcription factors, like Sox2, Sox5, Prrx1, Twist2, or Nr2f2 to overcome Yap dependency [61], which is associated with an EMT state that is different from classical TGF-beta-driven EMT. YAP-independent PDAC can be targeted with BETi, and therefore, the combination of BETi with YAPi exhibits synergism [61].

To physically connect promoter- and enhancer-associated proteins, promoter-enhancer interactions often require scaffolding proteins to assemble these factors located at different genomic regions. A frequently mutated scaffold protein, menin (MEN1), has been described as a tumor suppressor, and its inactivating mutations induce endocrine tumors including pancreatic neuroendocrine tumors (PanNETs). In PanNETs, nearly 40% of patients harbor *MEN1* mutations and it is the most commonly mutated gene [62, 63]. Additionally, reports show that non-mutated PanNETs often lack MEN1 expression [64, 65]. In this regard, a recent study identified the degradation of MEN1 via the E3 ligase Cullin4b. Inhibition of MEN1 neddylation or knockdown of CUL4 substrate receptor DCAF7 induced MEN1 protein accumulation and resulted in impeded malignant biological behaviors *in vitro* and reduced tumor volume *in vivo* [66]. In PDAC, on the other hand, *MEN1* is rarely mutated and seems to play a less important role in tumor development. Nevertheless, novel reports show that MEN1 interacts with histone deacetylases to repress CCAAT enhancer binding protein beta (C/EBPB) expression during TGF-beta signaling, inducing EMT and preventing C/EBPB-mediated tumor suppression [30].

Several attempts have been made to associate epigenetic patterns with PDAC subtypes [12, 40]. Lomberk et al. conducted an analysis of various chromatin states and linked these epigenetic clusters to the basal-like and classical PDAC subtype. Their study confirmed the alteration of specific enhancer and promoter regions in these subtypes. As anticipated, functional analysis of the epigenetic control regions demonstrated programs connected to pancreatic development and Ras signaling in the classical subtype. In basal-like PDAC, enhancer controlled programs for epidermal growth factor receptor (EGFR) and phosphatidylinositol 3-kinase (PI3K)-signaling as well as EMT-related pathways such as TGF-beta were detected. In addition, networks involved in cell differentiation and proliferation were linked to the epigenomic cluster matching basal-like PDACs [12, 40].

Apart from the modified epigenetic landscape in PDAC subtypes, a significant proportion of PDAC cases exhibit mutations in histone modifiers. A recent study revealed that the collective frequency of mutations in histone-modifying enzymes in PDAC patients surpasses 14%, emphasizing their importance (Fig. 2b) [7]. Several other studies have indicated a mutational frequency of up to 24% in various histone modifiers, with the most commonly mutated genes being the histone demethylase *KDM6A* as well as the histone methyl transferases *SETD2*, *KMT2C* (*MLL3*), *KMT2D* (*MLL3*) [6, 67, 68]. Other histone modifiers were less frequently affected (Fig. 2b). Beyond mutations, expression patterns of histone modifiers have also been intensively researched to decipher the complexity of epigenetic remodeling which has been linked to the tremendous cellular plasticity seen in PDAC [69–72]. In the following, we will discuss the impact of epigenetic modifiers that target the lysine residues of histones.

### Histone demethylases and histone methyltransferases

H3K27me3 is commonly linked to transcriptional repression whereas H3K4me3 is typically located at actively transcribed genes [75–77]. Active enhancer regions, on the other hand, are generally occupied by monomethylated H3K4 (H3K4me1) [78]. The most frequently mutated histone modifiers in PDAC are *KDM6A* as well as *KMT2D* (*MLL2*) and *KMT2C* (*MLL3*), which contribute to the H3K4 and H3K27 methylation landscape [6, 67, 79]. KDM6A, a Jumonji C domain demethylase also known as ubiquitously-transcribed X chromosome tetratricopeptide repeat protein (UTX), can orchestrate the demethylation of H3K27me3 with the destabilization of heterochromatin [80]. In pancreatic acinar cells, KDM6A acts in concert with the homeodomain transcription factor HNF1A to maintain the specific cell identity program [72]. In poorly differentiated cancer entities and PDAC metastasis, KDM6A displays a reduced expression [38, 67]. Moreover, it has been observed that *Kdm6a* loss accelerates tumor progression and metastasis development in genetically engineered PDAC mouse models, particularly in a gender-specific fashion [38, 67]. This phenomenon is attributed to the functional compensation by the Y chromosome-encoded UTY, which is a member of the KDM6 family but lacks demethylase activity. Mechanistically, the loss of KDM6A causes a demethylase-independent rewiring of the super-enhancer (SE) landscape, resulting in the enforced expression of drivers of dedifferentiation and metastasis, such as ΔNp63, MYC, RUNX3, or ZEB1 [38, 67]. The complex of proteins associated with set1 (COMPASS)-like complex contains the core methyltransferases KMT2C/D and KDM6A [81]. It was shown that the loss of KDM6A leads to an increase in the KMT2D and H3K4me1 signals at SE, which subsequently led to the proposal of a COMPASS-localization function that explains the rewiring of SE [38, 67]. In addition, activin A, a TGF-beta superfamily member, was shown to contribute to EMT via a non-canonical pathway involving the p38 mitogen-activated protein kinase in KDM6A-deficient cells [67].

Importantly, KDM6A-deficient PDACs expose specific therapeutic vulnerabilities. Elegant studies have observed that KDM6A instructs the tumor microenvironment (TME) of PDAC, whereby *Kdm6a*-deficient murine PDAC cells exhibit increased levels of the CXC motif chemokine ligand 1 (CXCL1) with subsequent recruitment of neutrophils, thus highlighting the CXCL1-CXCR2 axis as a potential therapeutic target [82]. From a tumor cell-intrinsic perspective, KDM6A is involved in regulating the mTORC1 signaling pathway in liver cancers and PDACs, presenting opportunities for the precise use of mTOR inhibitors [83]. Moreover, the loss of the KDM6A demethylase can be exploited using inhibitors of the BET family of proteins [38], as well as histone deacetylase inhibitors, as shown by previous studies [84].

On the opposite end of the H3K27 methylation spectrum, the histone methyltransferase EZH2 installs the repressive H3K27me3 mark. EZH2 is a member of the polycomb group (PcG) proteins, which are chromatin regulators organized in two complexes that are involved in gene silencing. Polycomb repressive complex 1 (PRC1) exposes E3 ubiquitin ligase activity to Lys118 and Lys119 of histone H2A (H2AK118ub and H2AK119ub), while the core polycomb repressive complex 2 (PRC2) contains EED, SUZ12, EZH2 (or EZH1), RBBP4, or RBBP7 [85]. The function of EZH2 is highly context-dependent, as exemplified in the carcinogenesis of the pancreas. In genetically engineered mouse models of PDAC driven by Kras^G12D^, the genetic inactivation of EZH2 accelerates early carcinogenesis due to a highly inflammatory microenvironment [86]. In contrast to the acceleration observed for early carcinogenesis, the inactivation of one allele of EZH2 results in deaccelerated PDAC development and impaired metastasis formation [70], underscoring the context- and stage-dependent functions of EZH2. One explanation for the context dependency is the crosstalk of EZH2 with the pro-inflammatory and pro-oncogenic transcription factor NFATc1. After a pancreatic injury, EZH2 represses the *NFATc1* gene, whereas, in established PDACs, EZH2 maintains NFATc1 expression [39]. Furthermore, the EZH2-dependent dedifferentiation was shown to be mediated by the transcriptional repression of transcription factors *GATA6* [70], which is a gatekeeper for epithelial differentiation [87]. Other contextual frameworks determining the functionality of EZH2 and relevant for the efficacy of EZH2 targeting, such as the mutational status of the tumor suppressor p53, were also shown to be important in PDAC [88]. In addition, recent data demonstrate how the methyltransferase cross-talks with the tumor microenvironment in established PDACs. EZH2 represses a pro-inflammatory transcriptome, contributing to profound immune suppression, with impaired NK and T cell surveillance, characteristics for PDAC [89].

The histone methyltransferases KMT2D and KMT2C regulate genomic regions by H3K4-monomethylation (H3K4me1) which is predominantly found at active enhancers co-marked with H3K27ac. These epigenetic genes are frequently mutated in PDACs. In a 2016 study using a PDAC mRNA expression dataset provided by the International Cancer Genome Consortium (ICGC), it was found that pancreatic cancer patients with low KMT2D and KMT2C mRNA expression had better survival rates [90]. Furthermore, a siRNA-dependent knockdown of *KMT2C* or *KMT2D* in human PDAC cell lines showed a proliferation-promoting role for both KMTs [90]. Opposingly, increased growth of PDAC cells was observed upon *KMT2*D knockdown *in vitro* and *in vivo*, with reduced expression of KMT2D being linked to metabolic rewiring involving an increased usage of aerobic glycolysis and polyunsaturated fatty acids (PUFAs) to satisfy the increased demands associated with proliferation [91]. Furthermore, the TGF-beta signaling pathway controls KMT2D expression, with metastatic PDAC revealing the lowest KMT2D protein expression [92]. This combined with the connection to the EMT regulator TGF-beta suggests a role for KMT2D in the metastatic cascade. Consistently, CRISPR/Cas9 knock-out of *KMT2D* in PDAC cell lines led to the upregulation of metastasis-promoting pathways such as MYC, EMT, or angiogenesis [92]. It was observed that KMT2D knock-out cells increase the expression of activin A, which drives EMT via non-canonical p38 MAPK signaling [92]. Moreover, the KMT2D-dependent instruction of the tumor microenvironment might be relevant in PDAC [93], suggesting that epigenetic therapies can tune the tumor cell-intrinsic to -extrinsic rheostat.

### Histone acetyltransferases

The epigenetic writer’s histone acetyltransferases (HATs) are responsible for promoting the accessibility of genes and genomic regions by histone acetylation and subsequent euchromatin formation. Some HATs display additional succinyl-transferase abilities and provide secondary regulatory mechanisms [94–97]. HATs can be grouped into the families of GNATs, MYST, as well as p300/CBP [98]. The most prominent HAT, E1A binding protein p300, has been well described due to its involvement in the regulation of global transcriptional regulation and its potential to target all histone subunits [99]. In pancreatic cancer, both tumor-promoting and tumor-suppressing functions have been described for p300. The occurrence of the *EP300* gene mutation in pancreatic cancer (Fig. 2b) is a hint towards its tumor-suppressive function. On the other hand, multiple studies described an increased activity of the proto-oncogene MYC mediated by p300 in other tumor entities [100, 101]. In a recent study, it was found that mutations in *EP300* lead to resistance against inhibitors of porcupine (PORCN), an endoplasmic reticulum O-acyltransferase, in RNF43-mutant pancreatic cancers [102]. PORCN is responsible for the palmitoylation of Wnts, which is necessary for their secretion and activation of the receptors [103, 104]. Approximately 5–10% of PDAC cases contain mutations in the RNF43 E3 ubiquitin ligase [6, 105], which can direct Wnt surface receptors to the proteasome [106, 107]. Therefore, PDACs with inactivated RNF43 express a higher level of Wnt receptors, such as Frizzleds (FZDs) and LRP5/6, making them more susceptible to inhibitors targeting ligand-activated Wnt signaling [108]. p300 plays a crucial role in the expression of GATA6, and the inactivation of *EP300* leads to dedifferentiation and a shift towards a more basal-like subtype. This dedifferentiation process, which bypasses WNT-dependent pathways, explains the resistance of *RNF43*/*EP300* double mutant PDACs to PORCN inhibitors [102].

Furthermore, elevated levels of miRNAs that target p300 and subsequent low expression of p300 were observed in PDAC cell lines displaying high metastatic potential in orthotropic mouse models [109]. Irrespective of metastatic potential, multiple studies have shown that inhibition of p300 and its coactivator CBP also improved gemcitabine sensitivity and reduced cell-cycle progression and proliferation in PDAC cells [110–113]. Shi et al. elaborated on this topic and observed a correlation between platelet-derived growth factor C (PDGFC) expression and gemcitabine resistance as a result of platelet-derived growth factor receptor alpha (PDGFRα) and beta (PDGFRβ) activation. They showed that p300 binds to the promoter site of the *PDGFC* gene, and inhibition of p300 reduced PDGFC expression [113]. Since PDGFRβ signaling has been linked to a more aggressive and metastatic phenotype in PDAC [114, 115], the observed regulation of PDGFC by p300 provides evidence for a more aggressive phenotype orchestrated by p300-dependent chromatin remodeling [113]. In a recent study, promising findings in mice regarding a novel dual inhibitor, XP-524, targeting BET proteins and p300 were presented. The results demonstrated improved survival and a reorganized microenvironment upon XP-524 treatment, resulting in enhanced immune infiltration [116]. Furthermore, the researchers noted that the inclusion of an anti-PD-1 antibody in the treatment regimen further enhanced survival. These findings suggest the potential of XP-524 as a therapeutic approach, possibly in combination with immune checkpoint inhibitors, for improved outcomes in the future [116]. Regarding other HATs, researchers discovered an increased expression of histone acetyltransferase 1 (HAT1) in pancreatic tumor tissue compared to healthy tissue, and high expression of HAT1 correlated with a worse prognosis [117]. The knockdown of *HAT1* resulted in reduced proliferation and tumor volume in the investigated human cell lines and murine models. The researchers additionally observed a correlation between HAT1 and PD-L1 expression and identified increased recruitment of BRD4 to the PD-L1 promoter in a HAT1-dependent manner. Whether HAT1 mediates BRD4 recruitment via H4K5 acetylation or its newly discovered succinyl-transferase activity remains unclear [94, 117]. Regardless, the results indicate that HAT1 might contribute to immune evasion during PDAC progression. Similar to HAT1, lysine acetyltransferase 2A (KAT2A/GCN5) also showed increased, aberrant expression in PDAC tissue and significantly worse overall survival in the KAT2A^high^ group in a cohort of 40 patients [97]. KAT2A knockdown resulted in reduced proliferation, impaired wound healing, and decreased invasion ability. Mechanistically, KAT2A was found to transcriptionally regulate YWHAZ (also known as 14-3-3ζ) through H3K79 succinylation [97]. 14-3-3ζ stabilizes β-catenin and maintains canonical Wnt signaling [118]. The knockdown of KAT2A led to reduced expression of 14-3-3ζ and subsequent degradation of β-catenin [97]. This, in turn, resulted in decreased expression of β-catenin target genes, including c-MYC, GLUT1, LDHA, and cyclin D1. Interestingly, the researchers attempted to restore the phenotype of KAT2A knockdown cells by reintroducing KAT2A^wt^. They observed successful reversion of the phenotype; however, when they reconstituted a succinyl-transferase defective form of KAT2A (KAT2A^Y645A^), they failed to restore the expression of 14-3-3ζ and H3K79 succinylation, despite the rescue of the H3K9 acetylation. These results emphasize the significance of acetylation-independent functions of KAT2A, particularly concerning the aggressiveness of PDAC [97].

Finally, Ono et al. analyzed immunohistochemistry staining of H3K9 and H3K27 acetylation in a cohort of 102 pancreatic cancer patients indicating a significantly worse overall survival in patients with intermediate staining compared to patients with high/low staining [111]. Considering that changes in H3K27ac precede enhancer reprogramming and are essential for cellular plasticity, it is not surprising that HATs may possess dual-functional roles in PDAC. The results of Ono et al. imply that preservation of a plastic acetylation state might lead to the worst prognosis and resembles the concept of the previously described p-EMT phenotype.

### Histone deacetylases

**Fig. 3:**
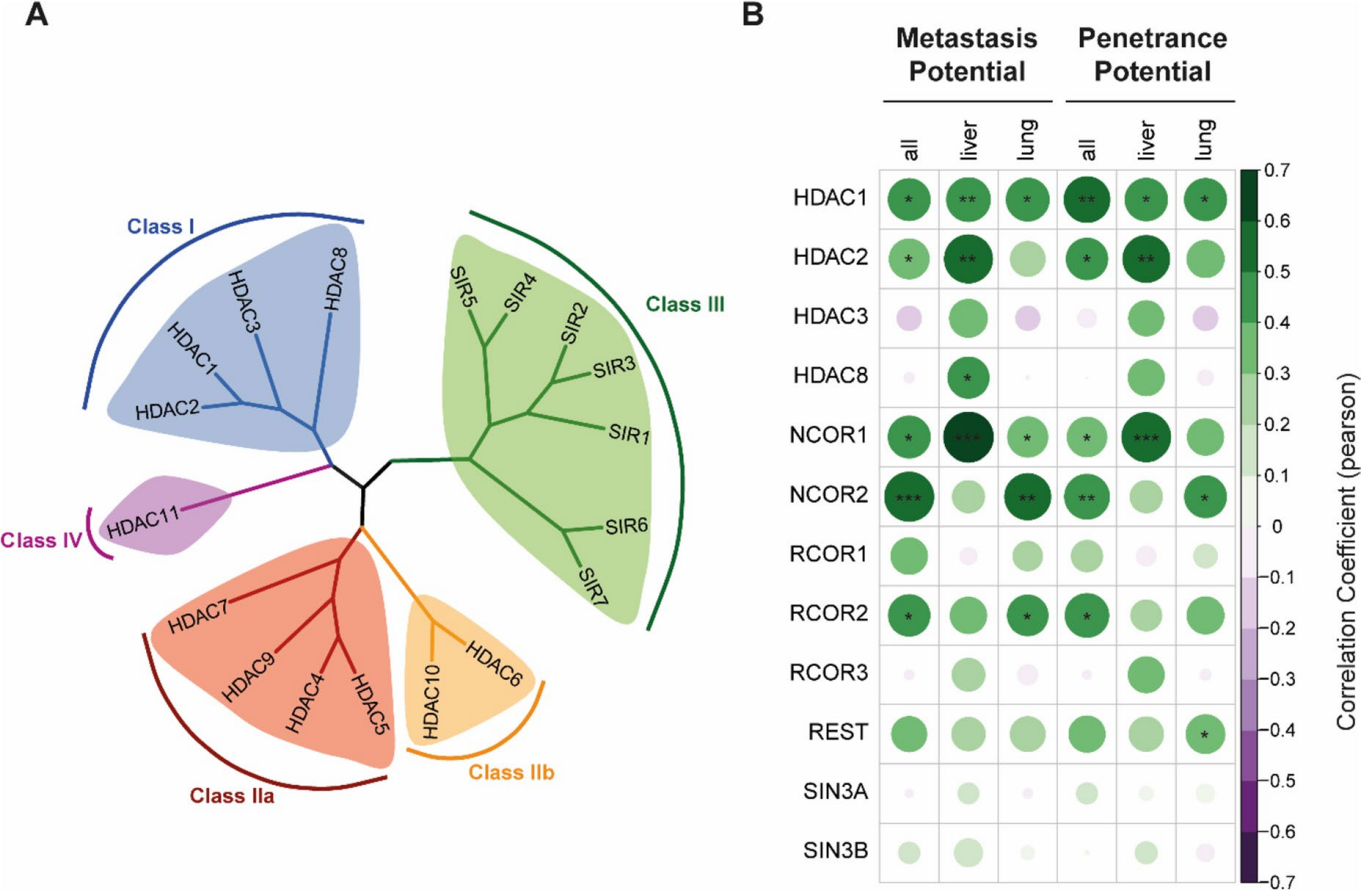
Histone deacetylases in PDAC. **a** Phylogenic tree of human HDACs. **b** Pearson correlation between metastatic/penetrance potential and RNA expression of class I HDACs and repressor complexes. Positive correlation is depicted in green and negative correlation is depicted in purple. *, *P*<0.05; **, *P*<0.01; ***, *P*<0.001. Amino acid sequences of human HDACs were downloaded from uniprot [136], and phylogenic tree was generated using simple phylogeny [137, 138] and ggtree [139]. Correlation coefficient data was downloaded from DepMap [140–142]

The cellular counterparts to HATs are histone deacetylases (HDACs). The family of HDACs has been well conserved across evolution. They are generally grouped into four different classes according to the sequence homologies with their corresponding yeast homolog [119, 120]. Classes I, II, and IV contain a Zn2+-ion in their enzymatic center whereas members of class III, also called sirtuins, are reliant on NAD+ as a cofactor and are therefore often regarded separately [121]. HDAC1-3 as well as HDAC8, belong to class I whereas HDAC4, 5, and 7 belong to class IIa, and HDAC6 and HDAC10 belong to class IIb respectively. HDAC11 is the sole identified member of class IV (Fig. 3a). HDACs primarily control gene transcription by deacetylation of the lysine residues at the histone tails causing condensation of the chromatin at the deacetylated regions which in turn prevents the binding of the transcriptional machinery [119, 122, 123]. The recruitment of HDACs to their designated target region on the genome is mainly coordinated by different repressor complexes. Those complexes include proteins with histone-recognition motifs like inhibitor of growth family member 2 (ING2) which recognizes and binds to H3K4me3 and can interact with transcription factors that require HDAC recruitment for their repressive functions [124, 125].

Although mutations of HDACs are not commonly found in PDAC, several studies have reported increased expression of HDACs in tumor tissue compared to normal pancreatic tissue [126–128]. Among the HDACs, class I HDACs, particularly HDAC1, HDAC2, and HDAC3, are highly represented [128]. Furthermore, previous studies have indicated that less differentiated tumors exhibit elevated expression of class I HDACs [127, 129]. A closer examination of the metastasis map [99–101], whose results are based on the implantation of cancer cell lines into mice and allows for the association of metastasis patterns with clinical and genomic characteristics, has confirmed a link between class I HDACs and repressor complexes with PDAC metastasis (Fig. 3b). While HDAC1 showed a correlation with overall metastatic potential in the 30 available PDAC cell lines, HDAC2 and HDAC8 expression was particularly associated with organ-specific metastasis to the liver. HDAC3 did not directly correlate with metastatic potential. However, the subunits of the major repressor complexes of class I HDACs, specifically the corepressor subunits RCOR2 (COREST2), NCOR1, and NCOR2 (SMRT), exhibited a strong correlation with overall metastasis potential in the PDAC metastasis map (Fig. 3b). NCOR1 and NCOR2 are mainly responsible for recruiting HDAC3 to facilitate histone deacetylation [130], while COREST recruits HDAC1 and HDAC2 [131].

The findings regarding the deacetylase-controlled metastatic potential align with a recently published study conducted on genetically defined murine models of autochthonous PDAC. The study revealed a significant reduction in metastasis formation in *Hdac2*-deficient murine PDACs [69]. In *Hdac2*-deficient cells, the expression of several tyrosine kinase receptors (RTKs), including PDGFRα and PDGFRβ, was found to be decreased [69]. RTKs, including the ones mentioned, likely play a crucial role in ensuring cell survival throughout the different stages of the metastatic cascade [114, 115]. Moreover, a correlation between HDAC2 and the TGF-beta signaling pathway was observed. In the absence of HDAC2, murine PDAC cells undergoing TGF-beta-driven EMT exhibited a significant increase in reactive oxygen species (ROS), ultimately leading to cell death. These findings suggest that HDAC2 plays a role in orchestrating a program to detoxify ROS and in limiting the tumor-suppressive effects of TGF-beta [69]. Furthermore, TGF-beta downstream regulation was partially impaired in *Hdac2*-deficient cells as evidenced by impaired repression of the epithelial marker E-cadherin (*Cdh1*) and SNAI1 upregulation [69], which is concordant with earlier reports [132] and observations that HDAC inhibitor (HDACi) Domatinostat (4SC-202) attenuated TGF-beta signaling, including reduced expression of the EMT transcription factor ZEB1, as well as induced upregulation of genes in a BRD4 and MYC-dependent manner [133].

Additional experimental evidence has highlighted the role of HDAC1 in PDAC metastasis. Treatment of PDAC cells with HDACis, such as DHOB and vorinostat, resulted in a reduction in their invasive potential. This was accompanied by a decrease in the expression of the EMT transcription factor SNAI1 [129]. Furthermore, a cohort of 103 PDAC patients categorized based on HDAC1 protein expression revealed that patients with high expression of this class I deacetylase exhibited significantly lower distant metastasis-free survival (DMFS) [129].

Supporting these results, in a pooled CRISPR screen in human PDAC cells, the correlation between HDAC1 overexpression and the induction of EMT genes, along with an increase in cell migration was described [134].

In summary, there is a clear association between HDAC1 and HDAC2 and the metastasis of PDAC, presenting an opportunity for therapeutic intervention. However, it should be noted that a study in melanoma cells observed an epigenetic silencing of invasiveness genes partly via HDAC2 [135]. Delineation of such contextual differences will increase the understanding of how HDACs direct metastasis.

## Development of epigenetic PDAC therapies

With advances in drug development and improved methods of on-target detection, the opportunities for novel-targeted treatments are vastly growing. Proteomic assays confirming target specificity have recently shown surprising results in HDACi specificity and show remarkable differences concerning HDACi potency when targeting HDACs within their designated repressor complexes [143].

To address target specificity and target accessibility, drugs with new mechanisms of action are on the rise. Protein degraders such as PROTACs might offer opportunities to target proteins more specifically than prior and will allow addressing functions independent of the enzymatic activity [144, 145]. A promising example in PDAC treatment is the progress made with KRAS mutant-specific degraders [145]. In parallel, efforts to develop novel degraders for epigenetic targets are also increasing [144]. So far, most clinically approved epigenetic drugs have found applications in non-solid tumors, such as DNA methyltransferase (DNMT) inhibitors 5-azacytidine and 5-Aza-2-deoxycytidine for the treatment of myelodysplastic syndrome [146, 147]. The HDACis vorinostat, romidepsin, and belinostat have been approved for the treatment of refractory cutaneous T cell lymphoma and refractory peripheral T cell lymphoma, respectively [148, 149]. Another HDACi, panobinostat, which had been approved for the treatment of multiple myeloma in 2015, has since been revoked by the FDA on March 2022 due to a lack of follow-up data, questioning the value of HDACis in the clinic. In 2020, however, the EZH2-inhibitor tazemetostat was approved for the treatment of advanced epithelioid sarcomas, as the first epigenetic drug with an application in solid tumors [150], underscoring the potential of epigenetic therapies.

In PDAC, epigenetic drugs have yet to be approved. Considering the potential benefits that have been described in preclinical studies, current clinical studies have shown increasing interest. Recent efforts observed that priming primary PDAC cells with a selection of epigenetic drugs sensitized the tested cell line to chemotherapeutic treatment [151]. However, the overall response across cell lines displayed a high heterogeneity concerning the individual drugs. To predict beneficial priming and chemotherapeutic therapies, the researchers generated transcriptomic predictor signatures [151]. This study exemplifies the rationale for epigenetic-chemotherapeutic combination therapies and their stratification strategies. Additionally, at present, there are a variety of studies ongoing or in the process of patient recruitment that focus on the treatment of PDAC patients with epigenetic drugs (Table 1). A phase-II clinical trial including the previously mentioned tazemetostat is currently being initiated and combines the EZH2 inhibitor with the immune therapeutic durvalumab (NCT04705818). This clinical trial might yield promising results, as recent studies connected EZH2 to an immune suppressive microenvironment [89]. DNMT inhibitor azacitidine is also being investigated in multiple studies that include immune checkpoint inhibitors and chemotherapy (NCT03264404, NCT04257448). DNMTs were shown to contribute to PDAC metastasis and their inhibition in other solid tumors-potentiated anti-tumor immune responses [152]. Another trial involving azacitidine has recently been completed that investigates tumor recurrence after tumor resection and adjuvant treatment (NCT01845805). Unfortunately, the study examiners did not observe a significant difference in relapse or overall survival between adjuvant azacitidine treatment or control groups that did not receive any additional treatment [153].

**Table 1:** Active and recently completed epigenetic clinical trials in pancreatic cancer

Drug name	Additional treatment	Target	Clinical trial	Status	Phase	Tumor stage
Entinostat (MS-275)	ZEN003694	HDAC class I	NCT05053971	Recruiting	Ib	Stages II–IV, multiple tumor entities
Entinostat (MS-275)	Nivolumab	HDAC class I	NCT03250273	Complete	II	Unresectable, metastatic
Romidepsin (istodax)	Nab-paclitaxel Gemcitabine Durvalumab Lenalidomide	HDAC class I	NCT04257448	Active, not recruiting	I/II	Metastatic, stage IV
Romidepsin	-	HDAC class I	NCT01638533	Active	I	Recurrent, stage III, stage IV, multiple tumor entities
Ivaltinostat (CG200745)	Gemcitabine, Erltinib	pan-HDAC	NCT02737228	Complete [155]	I/II	Unresectable, locally advanced, metastatic
Ivaltinostat (CG200745)	Capecitabine	pan-HDAC	NCT05249101	Recruiting	I/II	Locally advanced, metastatic
Vorinostat (SAHA)	Sorafenib, Gemcitabine, Radiation	HDAC class I/IIa	NCT02349867	Complete	I	Neoadjuvant, all stages
Vorinostat (SAHA)	52 drugs	HDAC class I/IIa	NCT03878524	Recruiting	Ib	Locally advanced, metastatic, multiple tumor entities
Panobinostat	52 drugs	pan-HDAC	NCT03878524	Recruiting	Ib	Locally advanced, metastatic, multiple tumor entities
Tazemetostat (EPZ-6438)	Durvalumab	EZH2	NCT04705818	Recruiting	II	Advanced
Azacitidine	Pembrolizumab	DNMT	NCT03264404	Active, not recruiting	II	Unresectable, metastatic
Azacitidine (CC-486)	-	DNMT	NCT01845805	Complete	II	Resected, node positive
Azacitidine	Nab-paclitaxel Gemcitabine Durvalumab Lenalidomide	DNMT	NCT04257448	Active, not recruiting	I/II	Metastatic, stage IV
Decitabine (dacogen)	-	DNMT	NCT05360264	Recruiting	II	Locally advanced, metastatic

Despite showing little to no success in solid tumors so far, a variety of HDACis have also been included in recent combination studies with other treatment regimens including BET inhibitors (NCT05053971), as well as checkpoint inhibitors (NCT03250273, NCT04257448) and classical chemotherapy (NCT02349867, NCT03878524, NCT02737228). These studies include both class I-specific HDACis such as entinostat and romidepsin but also inhibitors with a broader target range such as panobinostat and vorinostat as well as ivaltinostat. Another clinical trial included vorinostat treatment to the neoadjuvant radiotherapy regimen based on previous sensitization results (NCT02349867) [154]. The recently finished phase I/II trials combining ivaltinostat with gemcitabine and erlotinib treatment showed promising results regarding toxicity and patient survival. However, due to its single-arm design, results must be further validated (NCT02737228) [155]. The common side effects of epigenetic therapies are illustrated in Table 2. However, concepts to lower adverse effects, like sequential scheduling or using low doses below the maximally tolerated dose maintain efficacy in pre-clinical models exist [156–158] and therefore, such concepts offer clinical opportunities to reduce adverse effects.

**Table 2:** Adverse events caused by epigenetic therapy in solid tumors

Class	Drug name	Adverse events	Serious adverse events (Grade ≥ 3)	Clinical trial
HDAC inhibitors	Entinostat	Anemia, arthritis, fatigue, nausea, vomiting	Arthritis, colitis, renal tubular acidosis	NCT03250273
	Panobinostat	Anemia, diarrhea, fatigue, nausea, thrombocytopenia vomiting, weight loss	Diarrhea, thrombocytopenia	NCT01056601 [159]
	Romidepsin	Anemia, anorexia, asthenia, fatigue, nausea, thrombocytopenia, vomiting	Leucopenia, nausea	NCT00112463 [160]
	Vorinostat	Nausea, vomiting, diarrhea, fatigue, anemia, thrombocytopenia, hyponatremia, hyperglycemia	Lymphopenia, nausea	NCT00983268 [161]
EZH2 inhibitors	Tazemetostat	Fatigue, nausea, vomiting, decreased appetite headache	Anemia weight loss, pleural effusion	NCT02601950 [162]
DNMT inhibitors	Azacitidine	Appetite/weight loss, fatigue, diarrhea, nausea, anemia	Neutropenia, leukopenia	NCT01845805 [153]
	Decitabine	Anemia, nausea, nausea, increased alkaline phosphatase	Febrile neutropenia, lymphopenia, hyponatremia	NCT02847000 [163]

## Conclusion

As research technology continues to advance, therapeutic stratification strategies for cancer patients are undergoing significant changes. The advancements in multi-omics analysis are opening the door to a new era of treatment regimens and improved targeting of specific subgroups. However, the complex nature of cancer, including cellular plasticity, mainly controlled by transcriptional and epigenetic flexibility, remains challenging.

Previous studies have demonstrated that metastasizing cells rely heavily on their adaptive capabilities. As they progress through the metastatic cascade, they must quickly overcome various challenges, including starvation, migration, invasion, and survival in the bloodstream, before establishing themselves at a distant organ site. Therefore, it comes as no surprise that disseminating cells heavily depend on epigenetic regulators. However, it is important to note that chromatin dynamics are highly complex and only partially understood. Additionally, emerging research suggests that histone-modifying enzymes not only modify histones but also alter the functionality of other proteins. Consequently, gaining a more comprehensive understanding of the role of epigenetic regulators in tumor progression will be crucial for the successful and precise design of clinical studies.
